# Arthrotomy for the Relief of Pain

**Published:** 1899-05-27

**Authors:** 


### ARTHROTOMY FOR THE RELIEF OF PAIN.

At a recent meeting of the Harveian Society, Mr. Lockwood pointed out how important a means for the relief of pain in certain cases of joint disease we possess in the operation of arthrotomy. The cases to which he especially alluded were those of arthritis of the wrist-joint.

The various small bones which enter into the formation of this joint are closely held together by dorsal, palmar, and interosseous ligaments, and there is no loose capsule, so that any inflammatory effusion within the joint must soon acquire considerable tension. Moreover, the inflamed synovial membrane may easily be squeezed between the bones, which are with difficulty thrust apart, and these causes together no doubt lead to the intolerable pain which is caused by even a small effusion of fluid in this joint. In all the cases reported the performance of the operation was followed by immediate cessation of the pain. In the cases in which the disease appeared to be in the wrist-joint the operation consisted of a longitudinal incision about two and a half inches in length into the dorsum of the joint. The joint was washed out with perchloride of mercury solution, and a drainage tube was inserted. Incision of the joint for suppurative disease is of course another matter, but in these cases the operation was done purely for the relief of pain, and no pus was found in any of them. In one case the mischief appeared to be located in the lower radio-ulnar articulation, and the incision was made so as to open this joint alone, the rest of the wrist-joint being left intact.

